# Risk factors for implant failure in reverse oblique and transverse intertrochanteric fractures treated with proximal femoral nail antirotation (PFNA)

**DOI:** 10.1186/s13018-019-1414-4

**Published:** 2019-11-08

**Authors:** Youliang Hao, Zhishan Zhang, Fang Zhou, Hongquan Ji, Yun Tian, Yan Guo, Yang Lv, Zhongwei Yang, Guojin Hou

**Affiliations:** Department of Orthopedics, Peking University Third Hospital, No. 49, North Garden Rd., Haidian District, Beijing, 100191 China

**Keywords:** Risk factors, Implant failure, Reverse oblique intertrochanteric fractures, AO/OTA 31-A3 fractures, PFNA

## Abstract

**Background:**

The incidence of intertrochanteric hip fracture is expected to increase as the global population ages. It is one of the most important causes of mortality and morbidities in the geriatric population. The incidence of reverse oblique and transverse intertrochanteric (AO/OTA 31-A3) fractures is relatively low; however, the incidence of implant failure in AO/OTA 31-A3 fractures is relatively high compared with that in AO/OTA 31-A1 and A2 fractures. To date, the risk factors for implant failure in AO/OTA 31-A3 fractures treated with proximal femoral nail antirotation (PFNA) have remained ambiguous. The purpose of this study was to identify the predictive factors of implant failure in AO/OTA 31-A3 fractures treated with PFNA.

**Methods:**

The data of all patients who underwent surgery for trochanteric fractures at our institution between January 2006 and February 2018 were retrospectively reviewed. All AO/OTA 31-A3 fractures treated with PFNA were included. Logistic regression analysis of potential predictors of implant failure was performed. Potential predictors included age, sex, body mass index, fracture type, reduction method, status of posteromedial support and lateral femoral wall, reduction quality, tip-apex distance and position of the helical blade in the femoral head.

**Results:**

One hundred four (9.3%) patients with AO/OTA 31-A3 fractures were identified. Forty-five patients with AO/OTA 31-A3 fractures treated with PFNA were suitable for our study. Overall, implant failure occurred in six (13.3%) of forty-five patients. Multivariate analysis identified poor reduction quality (OR, 28.70; 95% CI, 1.91–431.88; *p* = 0.015) and loss of posteromedial support (OR, 18.98; 95% CI, 1.40–257.08; *p* = 0.027) as factors associated with implant failure.

**Conclusions:**

Poor reduction quality and loss of posteromedial support are predictors of implant failure in reverse oblique and transverse intertrochanteric fractures treated with PFNA.

## Background

The incidence of intertrochanteric hip fracture is expected to increase as the global population ages. It is one of the most important causes of mortality and morbidities in the geriatric population [1]. Reverse oblique and transverse intertrochanteric fractures are classified as AO/OTA 31-A3 according to the Orthopaedic Trauma Association classification system [2]. The major fracture line in AO/OTA 31-A1 and A2 fractures runs obliquely from the proximal greater trochanter to the distal lesser trochanter. However, AO/OTA 31-A3 fractures have the opposite configuration, with the major fracture line running from distolateral to proximomedial.

The incidence of AO/OTA 31-A3 fractures is relatively low, accounting for 5.3–23.5% of all trochanteric fractures [3–6]. However, the incidence of implant failure in AO/OTA 31-A3 fractures is relatively high compared with that in AO/OTA 31-A1 and A2 fractures [7, 8]. Implant failure has a major impact on mortality and morbidities among elderly individuals with intertrochanteric fractures [9]. Although many studies have focused on risk factors for implant failure in intertrochanteric fractures, most of these studies focused on unstable fractures [7, 10, 11]. Unstable fractures include some AO/OTA 31-A2 and A3 fractures; however, AO/OTA 31-A3 fractures have unique biomechanical characteristics distinct from other intertrochanteric fractures [2, 12]. Since the clinical introduction of proximal femoral nail antirotation (PFNA) by the AO group in 2004, this type of cephalomedullary nail has been commonly used in unstable intertrochanteric fractures. To the best of our knowledge, no studies have focused on the risk factors for implant failure in AO/OTA 31-A3 fractures treated with PFNA. Therefore, we retrospectively analysed AO/OTA 31-A3 fractures treated with PFNA in our hospital with the aim of identifying predictive factors of implant failure.

## Methods

### Patient data

The data of all patients who underwent surgery for trochanteric fractures at our institution between January 2006 and February 2018 were retrospectively reviewed. The study was approved by the Ethical Committee of Peking University Third Hospital. Radiological data were reviewed to identify AO/OTA 31-A3 fractures based on the AO classification [2]. All AO/OTA 31-A3 fractures treated with PFNA were included. Patients with pathological, open, or ipsilateral knee or ankle fractures were excluded. Patients who did not undergo a complete follow-up examination at 12 months postoperatively were excluded.

Patient demographics and clinical characteristics, such as sex, age at surgery, body mass index, mechanism of injury, American Society of Anesthesiologists (ASA) score, AO/OTA classification and reduction method, were retrieved from the medical records.

### Operative protocol

Five experienced orthopaedic surgeons performed all of the surgeries. All patients received standard preoperative antibiotic prophylaxis. Reduction and internal fixation were performed with the patients in a supine position on a fracture table with the use of an image intensifier. Patients then underwent routine surgical procedures for PFNA implantation according to the manufacturer’s protocol. Postoperatively, patients were given standard prophylaxis for deep vein thrombosis. Partial weight-bearing was initiated following the appearance of fracture healing on radiographs, and total weight-bearing began with clinical fracture healing. Follow-up evaluations were performed at 1, 3, 6 and 12 months after the surgery and yearly thereafter.

### Outcomes

Immediate postoperative radiographs were reviewed for reduction quality, status of posteromedial support, status of lateral femoral wall, tip-apex distance (TAD) and helical blade position in the femoral head.

The postoperative quality of fracture reduction was described as good, acceptable or poor, according to the modified criteria of Baumgaertner and Solberg [13] and Kim et al. [14] (Table 1).

**Table 1:** Quality of postoperative reduction

1. Alignment [13]	
a. Anteroposterior view: normal or slightly valgus neck-shaft angle	
b. Lateral view: less than 20 degrees of angulation	
2. Displacement of main fragments [14]	
a. Anteroposterior view: displacement less than the medial cortical thickness	
b. Lateral view: displacement less than the anterior cortical thickness	
Good, both criteria of alignment and both criteria of displacement	
Acceptable, both criteria of alignment and only one criterion of displacement	
Poor, only one or neither criterion of alignment or neither criterion of displacement	

**Fig. 1:**
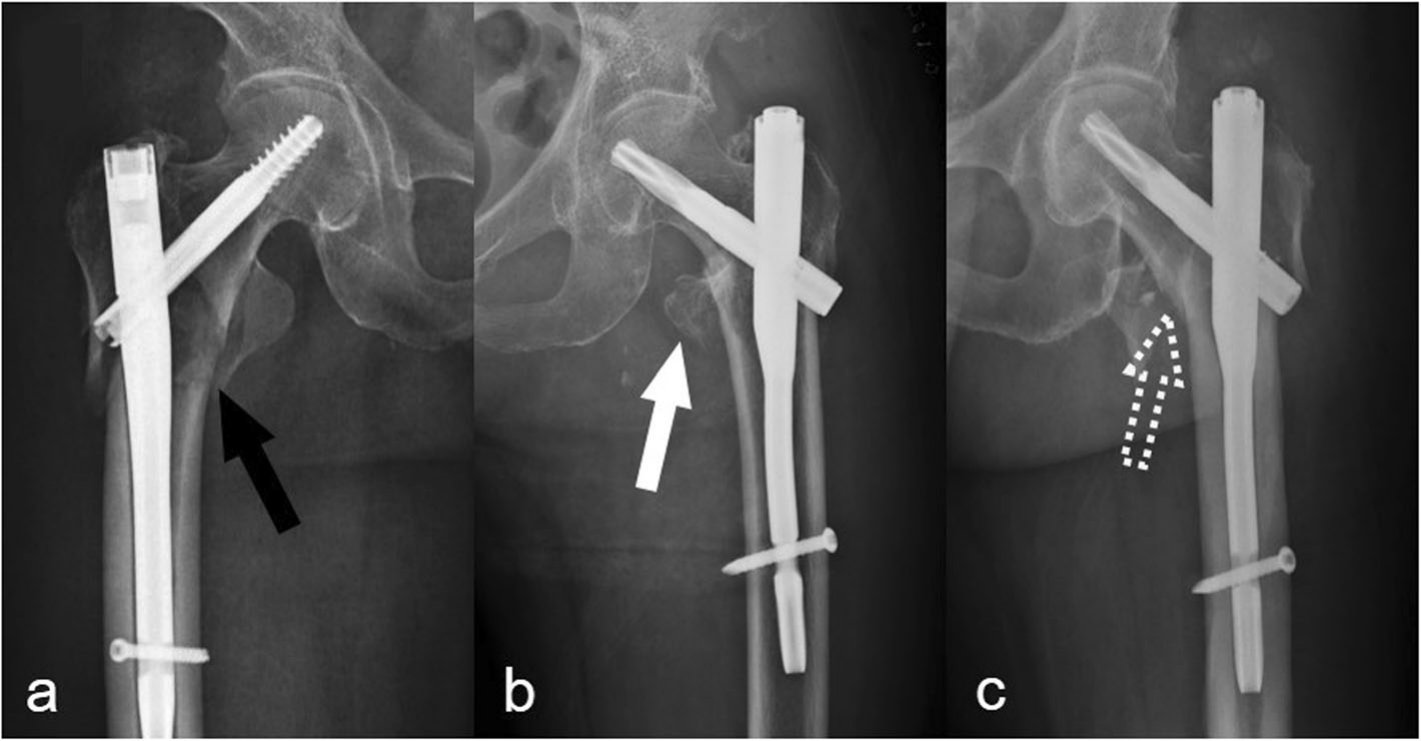
The status of posteromedial support. **a** A distinct absence of posteromedial support with no contact between the proximal and distal fragments (black arrow). **b** An avulsion fracture of the lesser trochanter with contact between the proximal and distal fragments (white arrow). **c** An impacted fracture of the proximal and distal fragments (white dotted arrow)

**Fig. 2:**
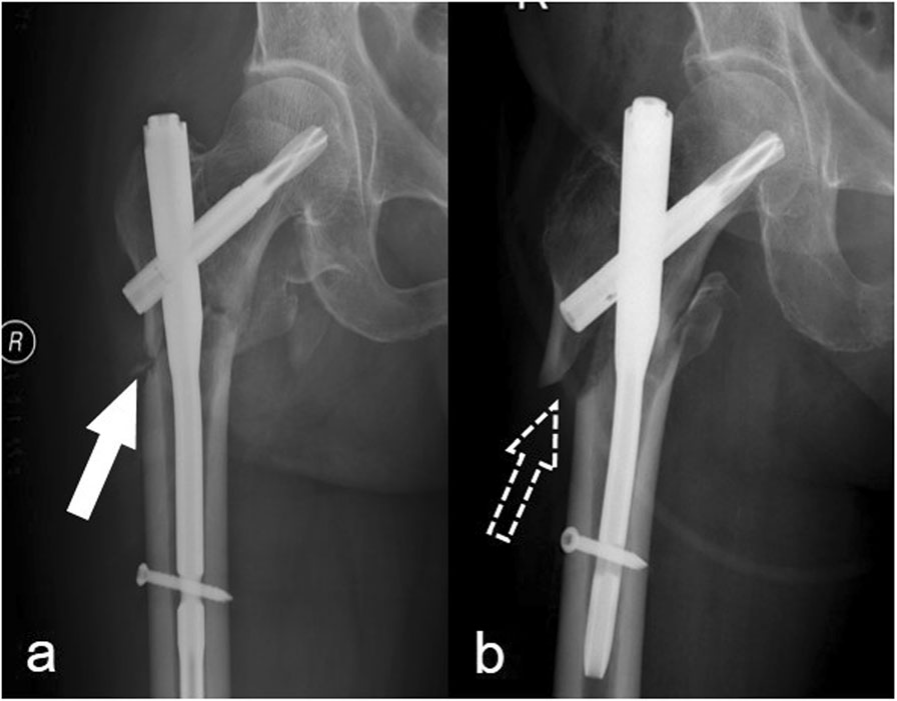
The status of the lateral femoral wall. **a** A reduced lateral femoral wall (white arrow). **b** A displaced lateral femoral wall (white dotted arrow)

The status of posteromedial support was defined as existence or loss according to the extent of displacement of the posteromedial fragment. A displacement of less than the cortical thickness implies that there is contact between the proximal and distal fragments, indicating the existence of posteromedial support. Otherwise, the status was recorded as loss of posteromedial support (Fig. 1a). The following features suggested the existence of posteromedial support: (1) avulsion fracture of the lesser trochanter (Fig. 1b) and (2) impacted fracture of the proximal and distal fragments (Fig. 1c).

The status of the lateral femoral wall was defined as reduced or displaced according to the extent of displacement. Good alignment and a displacement of less than the cortical thickness was defined as reduced (Fig. 2a). Otherwise, the status of the lateral femoral wall was recorded as displaced (Fig. 2b).

The TAD was determined by measuring the distance from the tip of the helical blade to the apex of the femoral head on both anteroposterior (AP) and lateral radiographs [13, 15]. The amount of radiographic magnification was determined precisely by the known diameter of the helical blade.

The position of the helical blade was recorded according to the zones described by Cleveland et al. [16] and subsequently used by Kyle et al. [17]. The femoral head was divided into superior, central and inferior thirds on the AP radiograph and anterior, central and posterior thirds on the lateral radiograph. Thus, nine zones were created to locate the helical blade position.

**Fig. 3:**
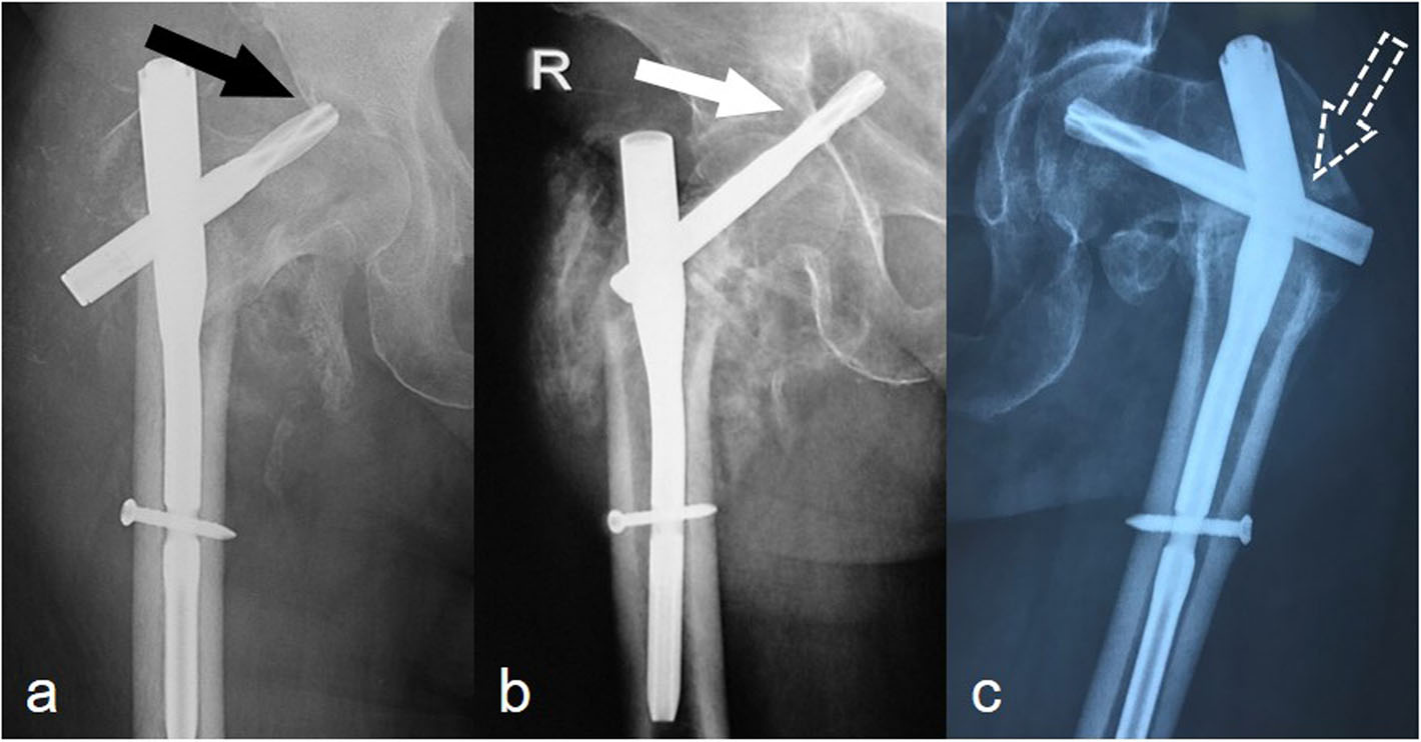
Types of implant failure. **a** Helical blade cutout (black arrow). **b** Helical blade perforation (white arrow). **c** Main nail breakage (white dotted arrow)

During follow-up, radiographs were reviewed for implant failure. Implant failure was defined as helical blade cutout or perforation and nail breakage. Cutout and perforation were defined as penetration of the helical blade from the femoral head into the surrounding soft tissues and hip joint, respectively (Fig. 3).

### Statistical analysis

The chi-squared test was used to analyse the distribution of categorical variables among comparison groups, and Student’s *t* test was used to analyse continuous variables. All variables were evaluated with an unconditional univariate logistic regression analysis. Odds ratios (ORs) and 95% confidence intervals (CIs) were obtained. A multivariate logistic regression model (enter methods) was designed to analyse the dependent variable ‘implant failure’ with a set of independent variables as risk factors. All test results were considered significant when *p* < 0.05, and all statistical analyses were performed using SPSS 22.0 (SPSS, Chicago, IL, USA).

## Results

Overall, 1124 consecutive trochanteric fracture patients underwent surgery at our institution between January 2006 and February 2018. One hundred four (9.3%) patients who had AO/OTA 31-A3 fractures were identified. Forty-nine patients were treated with PFNA. Two patients who did not complete the 12-month follow-up period were excluded. One patient who died from an internal disease within 1 month after the surgery was excluded. One polytrauma patient with an associated contralateral lower extremity fracture was excluded. Therefore, 45 patients who had A3 fractures treated with PFNA were suitable for our study.

There were 27 women (60%) and 18 men (40%) in our study. The mean age was 71.6 years (range, 19–92 years). Thirty-four patients were injured by a low-energy fall, seven patients were involved in a traffic accident, two patients fell from a height and two patients fell during skiing. Of these 45 patients, there were eight A3.1, seven A3.2 and thirty A3.3 fractures. The mean follow-up time was 26.6 months (range, 12–62 months).

Overall, implant failure occurred in six (13.3%) of forty-five patients; there were three cases of helical blade perforation, two cases of main nail breakage and one case of helical blade cutout.

Of the thirty-two cases of good reduction, implant failure occurred in three (9%), all three cases of which were helical blade perforation. Of the eight cases of acceptable reduction, implant failure occurred in none. Of the five cases of poor reduction, implant failure occurred in three (60%), there was one case of helical blade cutout and two cases of main nail breakage.

**Fig. 4:**
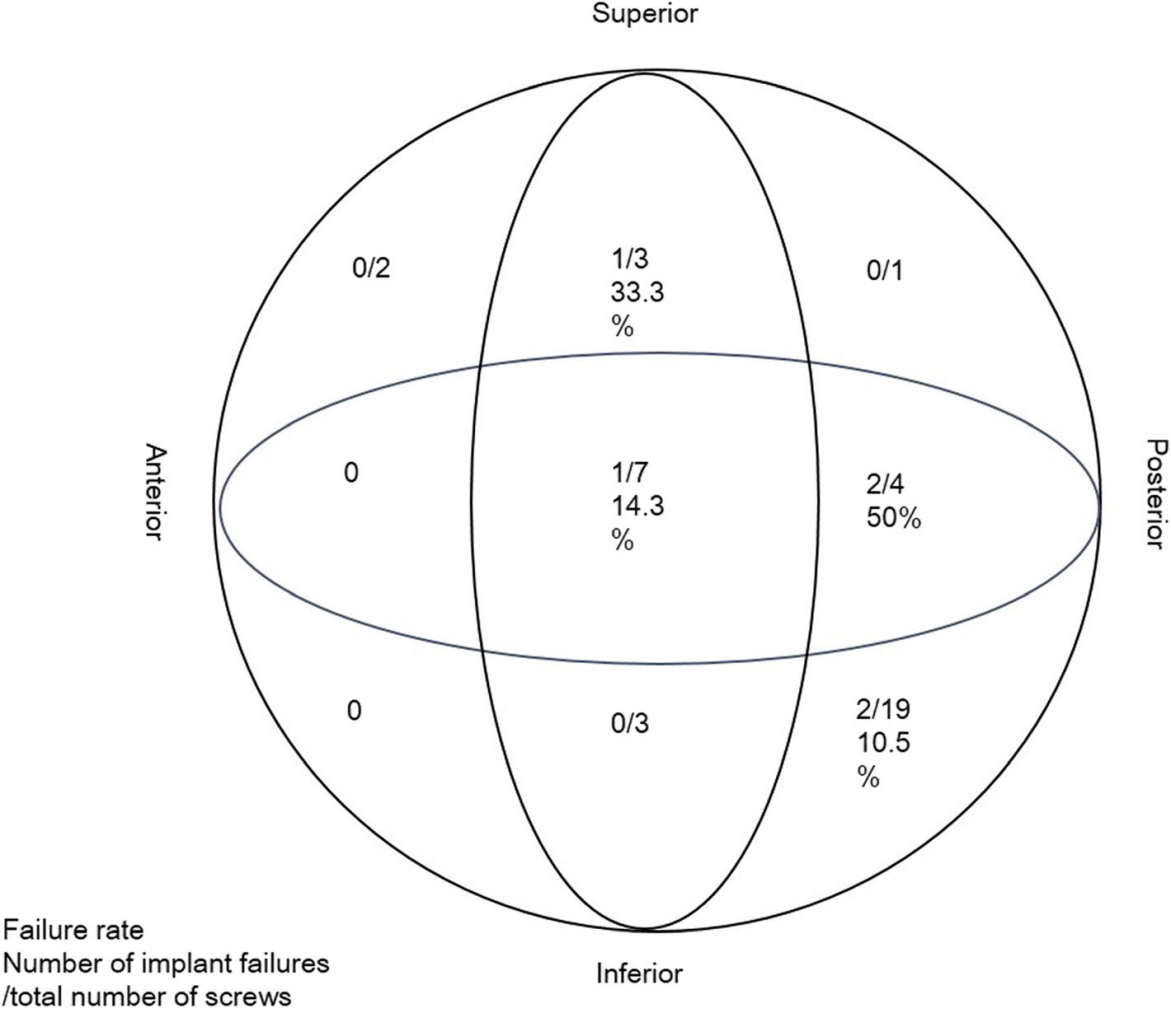
The distribution, by zone, of the 45 screws and of the six screws that failed. The number of implant failures in each zone is represented by the numerator, and the total number of screws in each zone is represented by the denominator. Failure rate: number of implant failures/total number of screws

Helical blades were found to have been placed in seven of the nine possible zones within the femoral head (Fig. 4). Among three blades placed in the superior-centre zone, there was breakage of one main nail (33.3%); among seven blades placed in the centre-centre zone, there was cutout of one blade (14.3%); among four blades placed in the centre-posterior zone, there was breakage of one main nail and cutout of one blade (50%); and among nineteen blades placed in the inferoposterior zone, there was perforation of two blades (10.5%). No cases of implant failure occurred in the remaining three zones.

Overall, the average TAD was 24 ± 6 mm. The mean TAD in the patients with and without implant failure was 22 mm and 24 mm, respectively (*p* = 0.52). The mean TAD in the patients with and without perforation was 19 mm and 24 mm, respectively. However, the difference was not significant (*p* = 0.19).

Initial univariate analysis identified poor reduction quality (OR, 14.50; 95% CI, 1.69–124.24; *p* = 0.015) and loss of posteromedial support (OR, 12.00; 95% CI, 1.65–87.52; *p* = 0.014) as factors associated with implant failure (Table 2).

**Table 2:** Univariate analysis of factors associated with implant failure (*n* = 45)

Parameter	Implant failure		OR (CI)	*p* value
	No, number (%)	Yes, number (%)		
Sex			0.63 (0.11–3.51)	0.593
Male	15 (38)	3 (50)		
Female	24 (62)	3 (50)		
Age			1.50 (0.16–14.56)	0.727
< 65 years	9 (23)	1 (17)		
≥ 65 years	30 (77)	5 (83)		
BMI				
< 18.5*	3 (8)	0 (0)	Undefined	
18.5–23.9	20 (51)	2 (33)		
≥ 24.0*	16 (41)	4 (67)	2.50 (0.41–15.43)	0.324
Mechanism of injury			0.58 (0.06–5.58)	0.637
Low energy	29 (74)	5 (83)		
High energy	10 (26)	1 (17)		
ASA score				
1	3 (8)	0 (0)	Undefined	
2	22 (56)	5 (83)	3.18 (0.34–30.16)	0.313
3	14 (36)	1 (17)		
AO/OTA classification				
31A-3.1	7 (18)	1 (17)		
31A-3.2	7 (18)	0 (0)	Undefined	
31A-3.3	25 (64)	5 (83)	1.40 (0.14–14.03)	0.775
Reduction method			2.11 (0.35–12.86)	0.420
Closed	20 (51)	2 (33)		
Open	19 (49)	4 (67)		
Lateral femoral wall			2.90 (0.50–16.76)	0.234
Reduced	29 (74)	3 (50)		
Displaced	10 (26)	3 (50)		
Reduction quality				
Poor	2 (5)	3 (50)	14.50 (1.69–124.24)	0.015
Acceptable	8 (21)	0 (0)	Undefined	
Good	29 (74)	3 (50)		
Posteromedial support			12.00 (1.65–87.52)	0.014
Existence	36 (92)	3 (50)		
Loss	3 (8)	3 (50)		

*BMI (body mass index); 18.5–23.9 as the reference value

After controlling for confounding variables, multivariate analysis still identified poor reduction quality (OR, 28.70; 95% CI, 1.91–431.88; *p* = 0.015) and loss of posteromedial support (OR, 18.98; 95% CI, 1.40–257.08; *p* = 0.027) as risk factors for implant failure (Table 3).

**Table 3:** Multivariate analysis of factors associated with implant failure (n = 45)

Parameter	OR	95% CI	*p* value
Poor reduction quality	28.70	1.91–431.88	0.015
Loss of posteromedial support	18.98	1.40–257.08	0.027

## Discussion

Although reverse oblique and transverse intertrochanteric (AO/OTA 31-A3) fractures were initially described some years ago [5, 18], their biomechanical characteristics, epidemiology and management criteria and the choice of implants are still being investigated [6, 19–22]. Biomechanically, AO/OTA 31-A3 fractures differ from standard intertrochanteric fractures. The incidence of AO/OTA 31-A3 fractures is relatively low; they account for 5.3–23.5% of all trochanteric fractures [3–6]. In our institution, AO/OTA 31-A3 fractures constituted 9.3% of all extracapsular proximal femoral fractures during the 12-year period we studied. The selection of an appropriate implant is critical for the fixation of these fractures, although the optimal treatment remains controversial. Studies have shown that compared with extramedullary devices, intramedullary nails could provide better stability and lower failure rates in AO/OTA 31-A3 fractures because the lever arm is relatively short and the nail prevents medial collapse of the distal fragment [4, 19, 22]. Various intramedullary nails have demonstrated a failure rate between 0 and 22% in previous studies [3, 6, 20, 23, 24]. In this study, the overall failure rate of PFNA was 13.3%. To the best of our knowledge, no studies have focused on the risk factors for implant failure in AO/OTA 31-A3 fractures treated with PFNA. Therefore, the aim of this study was to assess factors associated with implant failure after using PFNA to treat AO/OTA 31-A3 fractures.

Previous studies have shown that poor reduction of these fractures is associated with the occurrence of complications [8, 20]. Baumgaertner and Solberg [13] reported that with poor reduction instead of good reduction, fractures were more than three times more likely to progress to cutout. This study shows that there was an association between poor reduction and implant failure (OR, 28.70; 95% CI, 1.91–431.88; *p* = 0.015). Of the five cases of poor reduction, implant failure occurred in three (60%). However, the type of implant failure that occurred was different from that reported in previous studies. There was one case of helical blade cutout and two cases of the main nail breakage.

Posteromedial support has been reported as an important factor for the stability of intertrochanteric fractures [25, 26]. This study shows that loss of posteromedial support was a risk factor for implant failure in AO/OTA 31-A3 fractures treated with PFNA (OR, 18.98; 95% CI, 1.40–257.08; *p* = 0.027). Nevertheless, there are some different opinions about the role of posteromedial support in treating intertrochanteric fractures. A study conducted by Sharma et al. [27] concluded that neither fragmentation of the posteromedial fragment nor the size of the lesser trochanter fragment was found to predict stability in pertrochanteric fractures. However, the study involved only AO/OTA 31-A1 and A2 fractures, and the conclusion may not be applicable to AO/OTA 31-A3 fractures. Liu et al. [28] have also concluded that the integrity of the lesser trochanter has no significant influence on the surgical outcome of intramedullary nail internal fixation for intertrochanteric fractures. However, the study involved only three AO/OTA 31-A3 fractures. To the best of our knowledge, this is the first reported study on the influence of posteromedial support on implant failure in AO/OTA 31-A3 fractures.

The integrity of the lateral femoral wall has been identified as a prognostic factor in the healing of unstable intertrochanteric fractures. Proximal femoral fractures with a fracture of the lateral femoral wall have high rates of implant failure [29, 30]. Palm et al. [30] reported that patients with a fracture of the lateral femoral wall had a sevenfold greater risk of reoperation following dynamic hip screw fixation than did patients with an intact lateral femoral wall. In this study, we found that a displaced lateral femoral wall may increase the incidence of implant failure (OR, 2.90; 95% CI, 0.50–16.76; *p* = 0.234); however, the increase was not significant. We think that the sample size may not be sufficient, and further study of lateral femoral wall displacement is still needed.

Since Baumgaertner et al. [15] first reported the concept of the TAD, many studies have shown the importance of a proper TAD to avoid implant failure [7, 11, 13]. In Baumgartner’s study, fixation was accomplished with 142 extramedullary devices and 56 intramedullary devices. The results showed that no cutout occurred when the TAD was less than 25 mm [15]. Geller et al. [11] conducted another study, which included 82 pertrochanteric fractures treated with intramedullary devices, and they also suggested that surgeons should strive for a TAD of less than 25 mm when using intramedullary devices. However, in another study, 937 unilateral intertrochanteric fracture patients were enrolled, and all fractures were fixed with 135° dynamic hip screw devices. The authors suggested that the TAD should be kept to less than 15 mm [7]. In this study, the average TAD was 24 ± 6 mm. The mean TAD in those with and without implant failure was 22 mm and 24 mm, respectively (*p* = 0.52). The mean TAD of those with and without perforation was 19 mm and 24 mm, respectively. However, the difference was not significant (*p* = 0.19). In our opinion, the features of PFNA make the helical blade more likely to move axially than vertically, which means that if the helical blade is placed too deep, perforation may occur. Our opinion coincides with that of Zhou et al. [31], who have suggested that a TAD between 20 mm and 25 mm is optimal for helical blades. We believe that further research is needed to determine the best TAD for helical blades.

This study was associated with some limitations. First, the study was retrospective and was thus susceptible to missing data and bias. A further prospective study is necessary to reach more concrete conclusions. Second, the number of patients was relatively small, which may be due to the low incidence of AO/OTA 31-A3 fractures.

## Conclusion

In conclusion, the incidence of AO/OTA 31-A3 fractures is low; however, the incidence of implant failure in these fractures is relatively high. When using PFNA, it is important for surgeons to pay attention to fracture reduction, as poor reduction quality may increase the incidence of implant failure. Additionally, we found that loss of posteromedial support is another factor associated with implant failure in AO/OTA 31-A3 fractures treated with PFNA; however, further study is needed to determine whether the posteromedial fragment should be fixed.

## Data Availability

All data generated and analysed during this study are included in this article.

## Abbreviations

AP: Anteroposterior
ASA: American Society of Anesthesiologists
BMI: Body mass index
CI: Confidence interval
OR: Odds ratio
PFNA: Proximal femoral nail antirotation;
TAD: Tip-apex distance
